# Costs of Defense and a Test of the Carbon-Nutrient Balance and Growth-Differentiation Balance Hypotheses for Two Co-Occurring Classes of Plant Defense

**DOI:** 10.1371/journal.pone.0047554

**Published:** 2012-10-24

**Authors:** Tara Joy Massad, Lee A. Dyer, Gerardo Vega C.

**Affiliations:** 1 Department of Ecology and Evolutionary Biology, Tulane University, New Orleans, Louisiana, United States of America; 2 Department of Biology, University of Nevada, Reno, Nevada, United States of America; Centro de Investigación y de Estudios Avanzados, Mexico

## Abstract

One of the goals of chemical ecology is to assess costs of plant defenses. Intraspecific trade-offs between growth and defense are traditionally viewed in the context of the carbon-nutrient balance hypothesis (CNBH) and the growth-differentiation balance hypothesis (GDBH). Broadly, these hypotheses suggest that growth is limited by deficiencies in carbon or nitrogen while rates of photosynthesis remain unchanged, and the subsequent reduced growth results in the more abundant resource being invested in increased defense (mass-balance based allocation). The GDBH further predicts trade-offs in growth and defense should only be observed when resources are abundant. Most support for these hypotheses comes from work with phenolics. We examined trade-offs related to production of two classes of defenses, saponins (triterpenoids) and flavans (phenolics), in *Pentaclethra macroloba* (Fabaceae), an abundant tree in Costa Rican wet forests. We quantified physiological costs of plant defenses by measuring photosynthetic parameters (which are often assumed to be stable) in addition to biomass. *Pentaclethra macroloba* were grown in full sunlight or shade under three levels of nitrogen alone or with conspecific neighbors that could potentially alter nutrient availability via competition or facilitation. Biomass and photosynthesis were not affected by nitrogen or competition for seedlings in full sunlight, but they responded positively to nitrogen in shade-grown plants. The trade-off predicted by the GDBH between growth and metabolite production was only present between flavans and biomass in sun-grown plants (abundant resource conditions). Support was also only partial for the CNBH as flavans declined with nitrogen but saponins increased. This suggests saponin production should be considered in terms of detailed biosynthetic pathway models while phenolic production fits mass-balance based allocation models (such as the CNBH). Contrary to expectations based on the two defense hypotheses, trade-offs were found between defenses and photosynthesis, indicating that studies of plant defenses should include direct measures of physiological responses.

## Introduction

Herbivory and neighboring plant competition for resources are two of the most important biotic forces affecting plant distributions and fitness [1]. Competition, resource availability, and herbivory can affect levels of defensive compounds in plants, since chemical defense is a plastic response. Production of secondary metabolites is often associated with reduced fitness in terms of lower growth and reproduction [2]–[10]. This trade-off between investment in plant defense versus growth and reproduction is termed an allocation cost [10], [11]. However, comparisons between defense and growth or reproduction may be insufficient to quantify the costs of defense because natural selection may strongly favor reductions in trade-offs between such important activities as growth, reproduction, and defense. Physiological parameters can be more useful than growth rates for quantifying the cost of plant defenses [12]–[16], [8], [10] (but see [17]). Physiological costs, such as reductions in photosynthetic enzymes or the biosynthesis of other proteins required for primary metabolism are said to arise from ‘metabolic competition’ between defense production and primary metabolic functions [18]. Further examination of physiological costs is important for determining the mechanisms underlying allocation costs and for understanding interactions between pathways leading to primary and secondary metabolites. In addition, despite the notable contributions of induced defense literature to understanding costs of chemical defense, it may be particularly interesting to study costs in constitutive defenses to understand the baseline value plants place on tissue retention.

In terms of physiological costs, photosynthesis is among the most important variables to quantify as it forms the foundation of a plant’s carbon budget. Studies combining measures of plant defense and photosynthesis can also help clarify two prominent mass-balance based hypotheses of secondary metabolite production. The carbon-nutrient balance hypothesis (CNBH) [19] and the growth-differentiation balance hypothesis (GDBH) [11] were formulated to address differences in defense concentrations among individuals within a species; both hypotheses stem from the assumption that an imbalance in nutrients and carbon will allow plants to invest excess resources in defense as growth becomes limited before photosynthesis. Plants that produce nitrogen-containing defensive compounds (N-based defenses) are expected to increase their production of defenses when available nitrogen is more abundant than carbon; likewise, plants capable of synthesizing carbon-based secondary metabolites (C-based defenses) should increase production when fixed carbon exceeds requirements for growth [11], [19]. Nitrogen-rich enzymes and nitrogen-containing precursors are involved in the production of what are termed C-based defenses [20]–[23], however, so this classification of defenses as C- or N-based may be an oversimplification and confound interpretation of responses to resources in the framework of the CNBH or GDBH. There has, in fact, been much debate as to the utility of the CNBH [24],[25], and it has also been erroneously applied [26]. Nonetheless, the empirical support for this hypothesis shows predicted patterns of phenotypic changes in defenses for temperate woody [27], [28], herbaceous [29], and tropical [30]–[33] species.

The GDBH is more detailed than the CNBH and predicts a negative correlation between growth and defense under conditions of moderate to high resource availability [11]. The GDBH is difficult to test because: 1) a broad range of resource availability must be included in studies, 2) most variables assessed are merely correlates of the plastic physiological processes that are part of the hypothesis (e.g., biomass is often a proxy for resource allocation to growth, but it can include tissues and compounds important in defense and storage as well), and 3) it is difficult to ensure the maintenance of experimental resource conditions throughout a plant’s growth [34]. Despite these challenges, valuable insights on trade-offs and priorities in plant resource allocation can be gained from studies addressing aspects of the GDBH [35]–[37].

A key postulate of the CNBH and the GDBH is that defenses will increase under conditions of limited growth when photosynthesis continues to function at normal levels. This mechanistic aspect of the hypotheses is difficult to test, yet some studies have measured photosynthesis, growth, and defense simultaneously. Results from these studies show a variety of patterns. Light can increase photosynthesis and N-based defenses but decrease C-based defenses [38]; available nitrogen can increase photosynthesis and monoterpene production (except during the leaf expansion stage) [39], and high nitrogen can have inverse effects on photosynthesis (positive) and phenolic defenses (negative) [40], [41]. In addition, the down-regulation of genes important to photosynthesis has been shown to accompany herbivore induced up-regulation of defenses in *Nicotiana attenuata* (Solanaceae) [42], [43], although resource conditions mediate changes in transcription such that they do not always correspond to equivalent changes in the products encoded for [43]. Nevertheless, the paradigm persists that growth is more sensitive to a plant’s resource environment than is photosynthesis, and decreased growth with concomitant increases in defenses has been documented many times [11], [33], [44]–[47]. The sensitivity of photosynthesis to environmental conditions and the connection between photosynthesis and growth and defense production merit more empirical study.

Here we present experimental results quantifying saponin (terpenoid) and flavan (phenolic) production in a neotropical tree, *Pentaclethra macroloba* Kuntze (Fabaceae: Mimosoideae), a shade-tolerant species with nitrogen-fixing root nodules [48] that produces high levels of saponins which function as an antiherbivore defense [49], [50] as well as flavonoids. Saponins are a class of glycosylated triterpenoid, steroid, or steroidal alkaloid C-based compounds produced primarily via the mevalonic acid pathway [51], and flavans are flavonoids known to serve as plant defenses in a related genus, *Inga* [52], [53]. Most studies addressing the CNBH and GDBH have focused on phenolics [54], making studies of other classes of defense important. Terpenoids are especially interesting because they are produced by the mevalonic acid pathway, and defenses from this pathway do not fit predictions of the CNBH and GDBH as well as the phenolics produced via the shikimic acid pathway [20], [54]–[56].

We tested the hypothesis that saponin production in *P. macroloba* seedlings incurs both physiological (photosynthetic) and allocation (biomass) costs. We measured saponin and flavan production under different light regimes in response to changes in nutrients and plant density to test ecological predictions made by the CNBH and GDBH. Tests of the CNBH and GDBH have been criticized for not measuring complete costs of secondary defenses [57]; by quantifying relationships between two separate classes of defense, as well as photosynthesis and growth, we do not escape this criticism, but attempt to provide a more complete measure of these trade-offs.

## Materials and Methods

We collected seeds of *Pentaclethra macroloba* from multiple individuals distributed throughout the forest of La Tirimbina Rainforest Center, Sarapiqui, Heredia, Costa Rica (10° 23 N, 84°8 W) in January 2008. *Pentaclethra macroloba* was selected for this study because of its dominance in tropical forests where it is found [58], its diverse defensive chemistry, and the ease with which seeds can be found and propagated. La Tirimbina contains 345 ha of tropical wet forest (*sensu* [59]) with an average of 4000 mm annual precipitation, 26°C mean annual temperature, and an average day length of 12 hours.

### Planting Design

**Figure 1:**
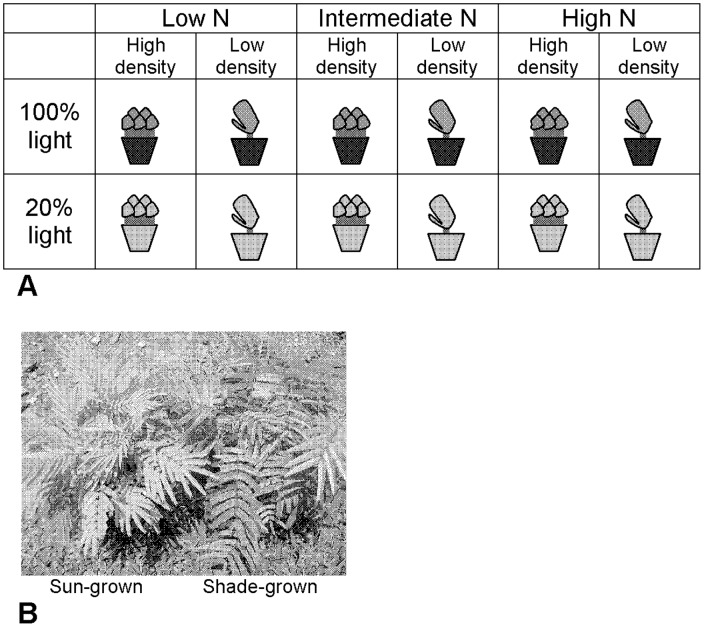
Schematic of experimental design. Seeds were planted individually or in competition with four conspecific neighbors and growth at low, intermediate, or high nitrogen levels. There were ten replicate pots per nitrogen x competition combination, five of which were grown in a shadehouse, and five of which were grown in full sunlight. The two light levels were analyzed as separate experiments (a). The photograph shows a sun-grown (left) and shade-grown plant (right) side by side (b).

One hundred eighty seeds were planted in 60 6-liter pots with 1.5 kg of sterile peat moss (Berger BM4: Sphagnum peat moss (coarse), dolomitic and calcitic lime, initial fertilizer charge, wetting agent; pH 5.4–6). A general fertilizer containing 25% phosphate, 41% potassium, 0.02% boron, 8.27% sulfur, 0.1% iron, 0.05% copper, 0.05% magnesium, 0.05% zinc, 0.001% molybdenum, and 25.459% inert ingredients (Miller Chemical and Fertilizer Corporation) was added to each pot at a concentration of 0.35 g/kg soil. Three levels of nitrogen fertilizer (urea: (NH_2_)_2_CO) were also applied: low = 0.002% N, intermediate = 0.004% N, and high = 0.008% N (20 pots per treatment). Seeds were planted alone (30 pots) and in competition pots (30 pots–5 seeds per pot). Half of the pots were then placed in full sunlight (∼1175 PAR (µmol/m^2^/s)) and half at 24% full sunlight (∼282 PAR) in a shadehouse at La Tirimbina (Figure 1). The plants in full sun were exposed to natural rain, and those in the shadehouse were watered regularly to ensure they received adequate moisture. The seedlings never appeared water-stressed. Only one shadehouse was available at the research station, so seedlings exposed to low light were grown together. Therefore, the two levels of the light treatment were analyzed as separate experiments to avoid pseudoreplication. Within each light regime, each combination of the nitrogen and competition treatments was replicated five times, with an individual pot as a replicate. Fertilizer was applied a second time in May 2008. Seedlings were routinely examined, and aboveground herbivore damage was not detected, so it is very unlikely that induction of defenses affected the data. Results indicate the competition treatment increased available nitrogen rather than decreasing it (because *P. macroloba* has N-fixing root nodules), and other work shows legumes can enhance the performance of neighboring plants [60].

### Seedling Measurements

Seedling height (cm), leaf area (cm^2^), the light saturated rate of photosynthesis (A_max_; µmol CO_2_/m^2^/s), and dark respiration (µmol CO_2_/m^2^/s) were measured for each replicate after six months of growth. Leaf samples were also collected at this time for chemistry analyses. The area of all the leaves on each seedling was measured as the length and width of the leaves multiplied together (cm^2^); the leaves are bipinnately compound, so this measurement was used to compare leaf sizes but not to determine actual leaf area. For pots with competition, the average height and leaf area of individuals in the same pot were used in analyses. Plant biomass was determined using regression equations from field collected seedlings (sun n = 10; shade n = 14). PAR at the seedlings was measured between 11∶00 and 13∶00, and the shade collected plants had an average PAR of 20% while the sun collected plants had an average PAR of 84%. The height and leaf area of the collected seedlings was measured, and the stems and leaves of the seedlings were then oven dried at 40 degrees Celsius for 72 hours and weighed. Regressions of aboveground biomass by stem height plus leaf area were then created (sun plants R^2^ = 0.76, *P* = 0.001; shade plants R^2^ = 0.55, *P* = 0.002). The resulting regression formulas were used to calculate aboveground biomass for the experimental seedlings.

A_max_ and dark respiration were measured with a LI-COR 6400 gas exchange system (LI-COR, Nebraska, USA), and only one individual was measured in pots with competition. The third leaf from the apical meristem was measured for consistency in leaf age. Measurements were made between 7∶00 and 13∶00 hours. Leaves were clamped into an airtight cuvette with a red-blue LED light source. Incoming CO_2_ was set to 380 µmol/mol from a CO_2_ cartridge. Light response curves were made from darkness to 10, 25, 50, 100, 150, 200 µmol/s and continued in increments of 200 µmol/s until an asymptote was reached. Leaves were given 120 seconds to adjust to each light level, and the CO_2_ differential was recorded when flow rate, CO_2_ and humidity were constant. The flow rate was set to 550 µmol/s, and humidity was between 65 and 75%. All necessary permits and permissions were obtained for the described field studies.

**Table 1:** MANOVA and profile analysis results for the response of *Pentaclethra macroloba* photosynthesis, biomass, and carbon-based metabolites (sugars, flavans, and saponins) to light, fertilizer, and competition.

Photosynthesis and biomass					
Factor	df	MANOVA F	*P*	Profile F	*P*
					
*Sun plants*					
Fertilizer	2	0.2	0.8	2.4	0.1
Competition	1	0.05	0.8	2.8	0.1
					
Error	23				
*Shade plants*					
Fertilizer	2	0.97	0.4	0.8	0.5
Competition	1	8.0	0.01	0.4	0.5
					
Error	23				
**Metabolites**					
**Factor**	**df**	**MANOVA F**	***P***	**Profile F**	***P***
*Sun plants*					
Fertilizer	2	0.2	0.8	0.2	0.8
Competition	1	19.5	0.0002	19.6	0.0002
					
Error	21				
*Shade plants*					
Fertilizer	2	0.8	0.5	1.1	0.4
Competition	1	0.2	0.6	0.03	0.9
Fert. * comp.	2	5.7	0.01	0.7	0.5
Error	17				

**Figure 2:**
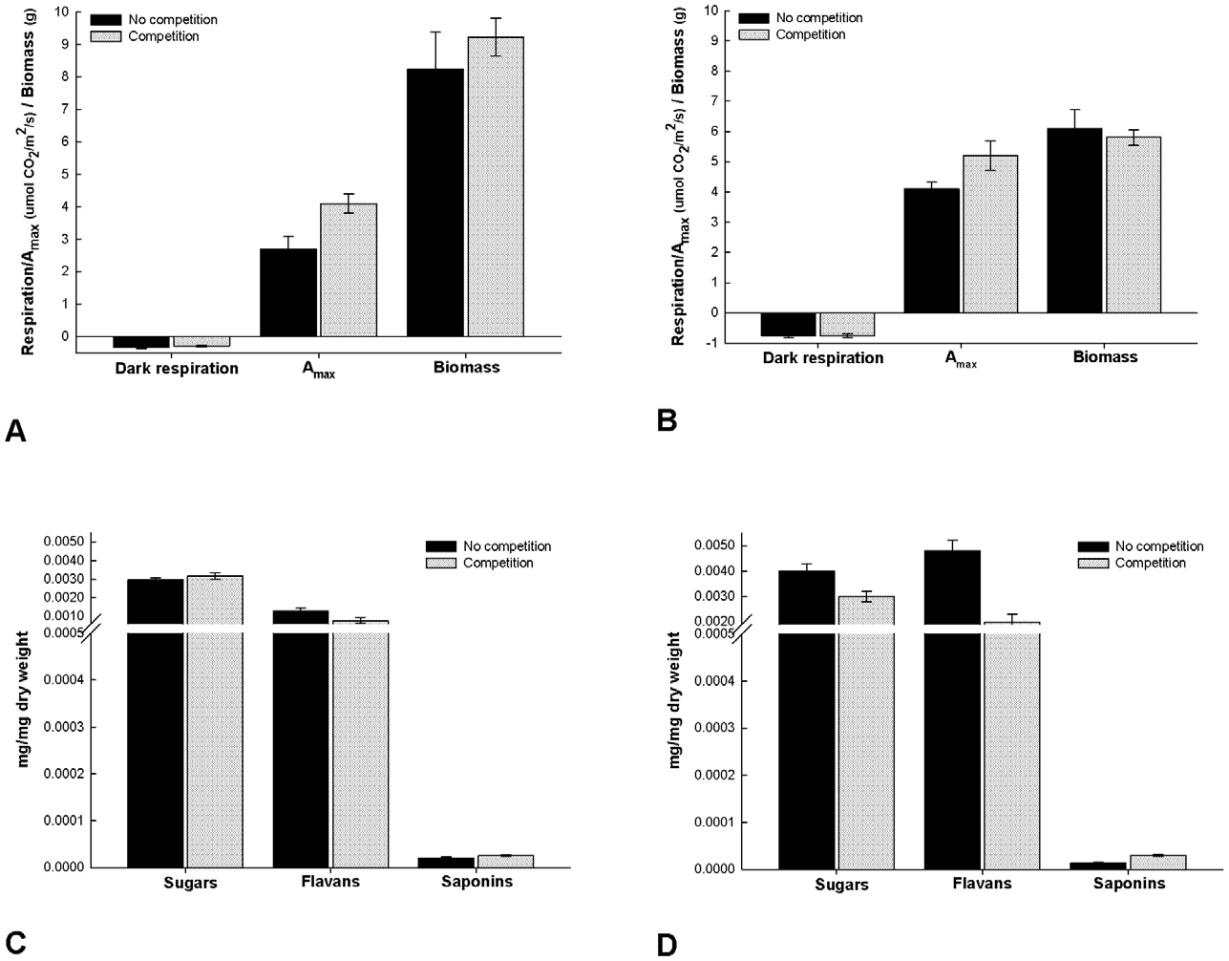
Means (SE) of photosynthesis, dark respiration, biomass, and carbon-based metabolites. Values are from *Pentaclethra macroloba* seedlings grown in shade (a, c) or full sunlight (b, d) with and without competition.

### Chemical Analyses

We collected and air-dried leaves for saponin and flavan quantification. In preparation for chemical extraction, leaf samples were dried overnight in an oven at low temperature and ground to a coarse powder. We utilized a new isolation and quantification procedure for saponin content [61]. One hundred milligrams of dry leaf powder were measured into a centrifuge tube and compounds were extracted from the leaf material in 30 ml of 80% ethanol with stirring. The samples were then centrifuged and the extracted compounds plus solvent were separated from the leaf material and dried. The process was repeated to extract any remaining compounds from the plant material. The dried samples were then dissolved in 15 ml methanol and defatted by shaking the solution with hexanes. The hexane layer was pipetted-off and the process was repeated. The defatted methanol layer was dried, and the samples were dissolved in 20 ml water. This solution was centrifuged to separate any remaining leaf material from the dissolved sample.

**Figure 3:**
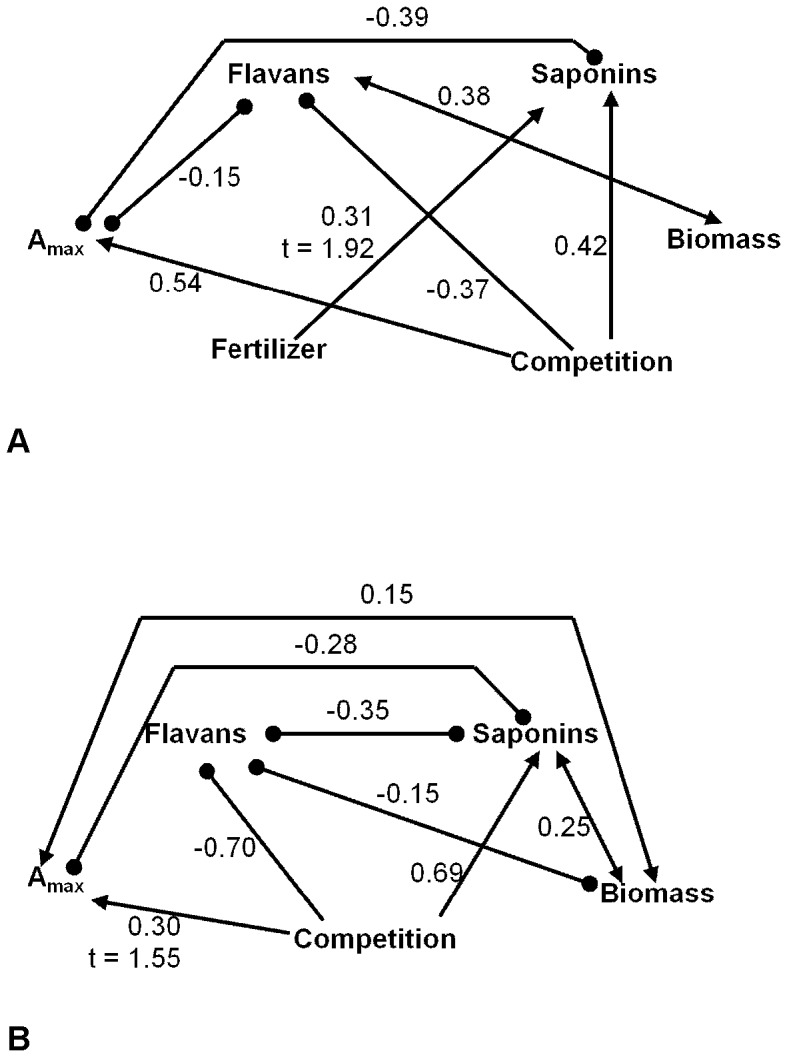
Interactions between experimental treatments and trade-offs in photosynthesis, growth, and defense production. Path diagrams showing causal relationships (single headed arrows) and correlations (double headed arrows) between competition, fertilizer, photosynthesis (A_max_), flavans, saponins, and biomass in *Pentaclethra macroloba* seedlings grown in the shade (A; χ^2^ = 0.8, df = 5, *P* = 0.98) or the sun (B; χ^2^ = 0.02, df = 1, *P* = 0.89). Bullets indicate negative relationships and arrows indicate positive relationships. Numbers are the standardized parameter estimates for relationships between variables. All relationships were significant with t-values >1.96 except where smaller t-values are indicated.

C-18 SepPak cartridges (Waters Corp., Massachusetts, USA) were then preconditioned with 15 ml acetone followed by 15 ml water. The water with dissolved sample was passed through the cartridge, and the elution was dried. The cartridge was then sequentially eluted with 20 ml each of 35%, 60%, and 100% methanol. The 35% and 60% methanol elutions were also dried. The 100% methanol elution was transferred directly to a pre-weighed scintillation vial and dried. The dried samples were redissolved in methanol, transferred to pre-weighed scintillation vials, and dried a final time. Samples were stored in the freezer. The water elution contains sugars and organic acids. The 35% elution contains flavans, the 60% layer is comprised of flavones, and the 100% layer contains saponins [61] (Lokvam, pers. comm.). Subsequent work demonstrated that the 60% elution contains mostly sapongenins (saponins without the attached polysaccharides), so it was not used in further flavonoid analyses. Samples were completely dried overnight in an oven at low temperature. Vials with samples were then weighed to determine the mass of each class of compound contained in the leaf material. The weights of samples from the elutions were used in analyses.

HPLC was utilized to examine five samples from the 100% elutions to confirm the presence of saponins and purity of the samples. The HPLC system consisted of a Hitachi LaChrom Elite HPLC System (Hitachi High Technologies America, California, USA) with a diode array detector (DAD; Hitachi High Technologies America) and an evaporative light scattering detector (ELSD; SEDEX 55 Evaporative Light-Scattering Detector; S.E.D.E.R.E., Alfortville, France). The column was packed with C8 coated beads (2×50 mm; 3 µm particle size; 100 Å pore size; Advanced Chromatography Systems, South Carolina, USA). Dry samples from the 100% methanol elution were dissolved in 250 µl 100% methanol and 10 µl were run on the HPLC at a gradient of MeOH:H_2_O (1∶1) to 100% methanol over 40 minutes with a 0.25 ml/min flow rate. The DAD absorption was set between 225–400 nm. The ELSD detector was maintained at 40°C and the pressure was at 2.3 bar. Nitrogen was the nebulizing gas.

### Statistical Analyses

Shade-grown plants and sun-grown plants were treated as two experiments and analyzed separately to avoid problems with pseudoreplication. Biomass and photosynthesis variables (A_max_ and dark respiration) were analyzed together using multivariate analysis of variance (MANOVA) followed by profile analysis with fertilizer, competition, and their interaction as independent variables; when interactions were not significant, main effects models were run. Primary (sugars) and secondary (flavans and saponins) metabolite production were likewise analyzed together with MANOVA and profile analysis. MANOVA analyzes the response of multiple dependent variables to experimental treatments, and profile analysis tests for differences in the magnitude or direction of response of different dependent variables. Three outliers were removed from dark respiration for the shade-grown plants for normality. A_max_ was square-root transformed for normality in the sun-grown plant dataset, and biomass was log-transformed in the sun dataset as well. Sugars and flavans were square-root transformed in both the shade and sun datasets.

We used structural equation models [62] to test hypothesized relationships between photosynthesis (A_max_), biomass, flavans, saponins, and planting treatments. Models were sequentially run testing *a priori* hypotheses of treatment effects and relationships between response variables, and the best fitting model is presented. The SAS Calis (Covariance Analysis of Linear Structural Equations) procedure was utilized to determine the fit of the models. The Calis procedure uses normal theory maximum likelihood procedures to estimate fit, and parameter vectors are estimated iteratively with a nonlinear optimization algorithm to optimize a goodness of fit function. Chi-square values are calculated for the maximum likelihood goodness of fit to determine the fit of the models. *P*-values greater than 0.05 indicate a good fit of the data to the model. We accepted the model with the highest *P*-value as the best description of the relationships between variables. All analyses were done with SAS 9.1 (SAS Institute Inc. 2003).

## Results

Photosynthesis and biomass of the shade-grown plants were highest with competition, but dark respiration was slightly higher without competition (Table 1; Figure 2a). The fertilizer treatment did not have an effect on the response variables. Neither photosynthesis, respiration, nor biomass of plants grown in the sun changed with the competition or fertilizer treatments (Table 1; Figure 2b).

The interaction between fertilizer and competition was significant for shade-plant metabolite production (Table 1). Sugars were higher in plants with competition and low or intermediate levels of fertilizer. They were lowest also with competition but with high levels of fertilizer. The two groups of secondary metabolites responded in the opposite direction to increased nitrogen. Flavans were highest in low nitrogen conditions (no competition, low fertilizer) and lowest in conditions of high nitrogen (competition and high fertilizer levels). In contrast, saponins were highest with competition and high fertilizer levels and lowest without competition and with low fertilizer levels (Figure 2c).

Metabolites of sun-grown plants were affected by competition such that sugars and flavans were all higher without competition (low nitrogen), and saponins were higher with competition (Table 1; Figure 2d).

The best-fitting structural equation models differed for plants grown in the sun or the shade (Figure 3). In both datasets, competition increased A_max_ and saponins, but decreased levels of flavans. Higher levels of nitrogen in the competition treatment likely allowed for increased photosynthesis and saponin production by providing nitrogen necessary for enzymatic processes. This increase in N may have instigated a diversion of C from flavan production to processes or pools that were limited under lower N conditions (negative effect of competition on flavans). Competition did not have a direct effect on biomass, however. The effect of competition in the sun was not quite significant for A_max_ (t-value of relationship = 1.55; a significant relationship is described by a t-value ≥1.96), but the best-fitting model included this pathway. The best-fitting model for shade-grown plants also included a slightly non-significant causal pathway indicating fertilizer increased saponin levels (t-value = 1.92). Both sun and shade plants showed evidence of a trade-off between photosynthesis and saponin production, and a negative relationship was also present between A_max_ and flavans in shade-grown plants. Flavans in shade-grown plants were also positively correlated with biomass, contrary to expectations of a growth-defense trade-off. Plants grown in the sun showed the opposite pattern, and flavans and biomass were negatively correlated. Perhaps full sunlight promoted increased growth (positive relationship between photosynthesis and biomass), creating demands on pathways of plant allocation that limited production of flavans. When the correlation between A_max_ and biomass was included in the model for shade-grown plants, the relationship was negative, although weak (PE = −0.02), and the model fit less well. The shade plant model including the relationship between saponins and biomass also fit less well, and the correlation between saponins and biomass was negative (PE = −0.07).

In summary, in sun-grown plant photosynthesis and biomass were positively correlated as were saponins and biomass. In the shade, both these relationships were negative. In contrast, flavans and biomass were negatively related in the sun, and their relationship was positive in the shade. Light therefore seems to be the limiting factor which, when abundant, allows for positive relationships between saponins and biomass and A_max_ and biomass or, when restricted, leads to a situation in which trade-offs between these processes and pools become apparent. Flavans, however, show a trade-off with growth only under full sun conditions.

## Discussion

Trade-offs between growth and defense differed with the light conditions seedlings were grown under, and the GDBH [11] and CNHB [18] were supported only by comparisons between flavans and biomass. The GDBH predicts growth and defenses will be positively correlated when resources are limited and negatively correlated when resources are abundant. As expected, we found that growth and defense were positively correlated in the shade, while the predicted trade-off between flavans and biomass became apparent in the sun. Both the CNBH and the GDBH assume that growth is limited before photosynthesis, allowing excess resources to accumulate and serve in defense production. By measuring photosynthesis, growth, and two classes of defense, however, we uncovered trade-offs between photosynthesis and defense when biomass and defenses were positively correlated. A similar trade-off between defense and photosynthesis rather than defense and growth was found for an imide (a N-based defense) in *Piper cenocladum* [63]. This is consistent with the hypothesis that costs of defense are not only manifested in growth and reproduction but exist at a physiological level. One important caveat is that we do not have data on root biomass. Overall results may change with the inclusion of information on allocation to below-ground growth; however, because *P. macroloba* have N-fixing root nodules and were grown in pots, differences in below-ground biomass were probably minimal.

The correlations between secondary metabolites and biomass suggest that flavans or saponins are not costly to a plant, except under conditions of full sunlight (contrary to expectations, costs should be most evident when resources are limited) [47]. However, including physiological data showed relationships between defenses and photosynthesis were negative under both shade and full sun. The trade-off between photosynthesis and defense production may occur because defense production, regardless of a compound’s classification as C- or N-based, requires nitrogen for enzymes involved in the metabolic pathways. The majority of nitrogen in a plant is contained in Rubisco, the primary enzyme in photosynthesis, which accounts for roughly 25% of leaf nitrogen in C_3_ plants. Rubisco content increases with leaf nitrogen and is sometimes, but not always, produced in excess of photosynthetic requirements as a means of nitrogen storage [64]. It is therefore possible that leaf nitrogen in *P. macroloba* is sufficiently limited, such that trade-offs between different cellular demands for nitrogen exist. In addition, flavan production decreased with presence of neighboring plants (higher nitrogen), following predictions of the CNBH and the GDBH that investments in C-based defenses decline as nitrogen availability increases.

In the shade experiment, a positive correlation between growth and defense was present for flavans. This relationship changed in the full sun, and flavans were negatively correlated with biomass. Initially, it would seem the negative relationship is due to increased growth at high light. Biomass was greater, however, in shade grown plants while dark respiration and photosynthesis were higher in the sun. *Pentaclethra macroloba* is a shade-adapted plant, so the increase in respiration may result from metabolic processes necessary to avoid photoinhibition, and the trade-off with biomass may be related to an underlying relationship with respiration. Including dark respiration in the structural equation model for sun-grown plants yielded a significant model (χ^2^ = 6.0, df = 3, *P* = 0.1), and flavans and respiration were negatively related while biomass and respiration were positively correlated. Flavan levels were at their highest in plants grown with full sunlight and no competition; this increased production could result from a greater need for the defensive role of flavonoids as UV-B protectants (e.g., [65]), and flavonoid production increases in full sunlight in other species as well [66].

Unlike flavans, triterpenoid saponin levels did not fit predictions of the two defense hypotheses, increasing with nitrogen and having a positive relationship with biomass. Phenolics are the class of secondary metabolites most often found to fit predictions of the CNBH [17], [54], [67]–[69], and it has been suggested that the CNBH and GDBH are more relevant to phenolics because they are produced via the shikimic acid pathway which competes directly with protein synthesis (growth) for nitrogen via metabolism of phenylalanine [54], [70], while terpenoids are produced by different biosynthetic pathways. Biosynthesis of saponins is initiated via the mevalonic acid and methylerythritol phosphate pathways [51], [70], which do not experience a direct trade-off with growth based on available nitrogen [71], [72]. Our data suggest saponins and photosynthesis compete for nitrogen before carbon is divided between growth and ‘excess’ carbohydrates (*as per* [54]). This may explain why fewer data from terpenoid studies fit predictions of the CNBH and GDBH.

Gershenzon speculated that the CNBH would apply to terpenoids only when they are substrate limited [20], but our data suggest saponin production was more limited by nitrogen resources required for synthesis rather than carbon required as a substrate, and this was also true in the shade for flavans. Overall, we found restricted support for the GDBH and the CNBH but have demonstrated that investigations of costs of defense should focus on the physiological level where many trade-offs appear to take place. In spite of context dependent support of the GDBH and CNBH based on terpenoids and phenolics, the appropriate application of these hypotheses should continue to guide experiments that enhance a clear understanding of plant defensive investments. Basic and applied ecology will benefit from advances in studies that document costs of defense against parasites, and further investigations of interactions between resource availability and physiological trade-offs will demonstrate the strength of both ecological and evolutionary influences on investments in defense–issues of particular contemporary importance due to rapid changes in carbon and nitrogen availability in the environment.
